# How Structural Features Define Biogenesis and Function of Human Telomerase RNA Primary Transcript

**DOI:** 10.3390/biomedicines10071650

**Published:** 2022-07-08

**Authors:** Maria Rubtsova, Olga Dontsova

**Affiliations:** 1 Department of Chemistry, A.N. Belozersky Institute of Physico Chemical Biology, Lomonosov Moscow State University, 119991 Moscow, Russia; olga.a.dontsova@gmail.com; 2 Shemyakin-Ovchinnikov Institute of Bioorganic Chemistry of the Russian Academy of Sciences, 117997 Moscow, Russia; 3 Center of Life Sciences, Skolkovo Institute of Science and Technology, Skolkovo, 121205 Moscow, Russia

**Keywords:** telomerase RNA, RNA biogenesis, RNA processing, localization, alternative function, transport, *hTERP*

## Abstract

Telomerase RNA has been uncovered as a component of the telomerase enzyme, which acts as a reverse transcriptase and maintains the length of telomeres in proliferated eukaryotic cells. Telomerase RNA is considered to have major functions as a template for telomeric repeat synthesis and as a structural scaffold for telomerase. However, investigations of its biogenesis and turnover, as well as structural data, have provided evidence of functions of telomerase RNA that are not associated with telomerase activity. The primary transcript produced from the human telomerase RNA gene encodes for the *hTERP* protein, which presents regulatory functions related to autophagy, cellular proliferation, and metabolism. This review focuses on the specific features relating to the biogenesis and structure of human telomerase RNA that support the existence of an isoform suitable for functioning as an mRNA. We believe that further investigation into human telomerase RNA biogenesis mechanisms will provide more levels for manipulating cellular homeostasis, survival, and transformation mechanisms, and may contribute to a deeper understanding of the mechanisms of aging.

## 1. Introduction

Telomerase is a ribonucleoprotein complex that is essential for telomere maintenance [1,2]. Telomeres are special structures located at the ends of linear eukaryotic chromosomes which are shortened in each round of cellular division due to the end replication problem and nuclease action. Critically short telomeres appear after a particular number of cellular divisions (50–70), activating the cellular senescence mechanism, which results in death in a majority of cells; however, some cells may overcome this crisis, acquire the ability of unlimited proliferation associated with cancer phenotypes, and activate telomerase as a major mechanism to maintain telomere length. Telomerase is active in limited types of cells, and its functioning has been associated with the proliferative status of cells [3,4]. For example, embryonic, stem, germ, cancer cells, activated immune cells, cells participating in regenerative processes, and other cells with a high proliferation level present telomerase activity. The decreased capacity to activate telomerase during regeneration and the immune response are associated with the aging of organisms. In contrast, the activation of telomerase in cells with uncontrolled proliferation stimulates oncogenesis. 

Telomerase contains two core components—telomerase reverse transcriptase (TERT) and the telomerase RNA component (TERC)—which are necessary for telomere synthesis [5]. To present telomerase activity, cells should express both of these core components of telomerase (i.e., TERT and TERC) [6,7,8]. *hTERT* expression is tightly regulated and, as a rule, the expression level of *hTERT* determines the level of telomerase activity. A majority of somatic cells lose telomerase activity through the silencing of *hTERT* gene expression during embryogenesis [9,10]; however, the expression of the *hTERC* gene remains constant in the majority of somatic cells [6]. It is not silenced and may be increased to facilitate telomerase activity in proliferated cells. The non-canonical non-telomeric functions of both core components of human telomerase have recently been reviewed [11]. The *hTERC* protein coding potential in the synthesis of human telomerase RNA protein (*hTERP*) has recently been uncovered [12]. *hTERP* is important for cell survival under stress conditions [12] and has been shown to be involved in autophagy regulation [13]. Although the existence of *hTERP* has been demonstrated, the canonical tertiary structure of *hTERC* described for the telomerase complex is not convenient for ribosome binding and translation initiation. Unfortunately, *hTERP* properties and functions have not been deeply investigated at this time and we do not know the correlation of *hTERP* appearance with the expression of *hTERT* and *hTERC.* However, we hypothesize that *hTERP* is synthesized when hTERT is absent in cells. *hTERP* may be constitutively expressed, like *hTERC*, and the presence of hTERP may be important for alternative telomere maintenance mechanism realization. The expression profile of *hTERP* and its correlation with the proliferative status of cells as well as with metabolism should be investigated in future. In this review, we discuss the alternative structural features of transcripts derived from the *hTERC* gene, which must define whether telomerase RNA acts as a telomerase component or as mRNA encoded for *hTERP*. 

## 2. Diverse Forms of Human Telomerase RNA Are Processed from the Nascent Transcript

**Figure 1:**
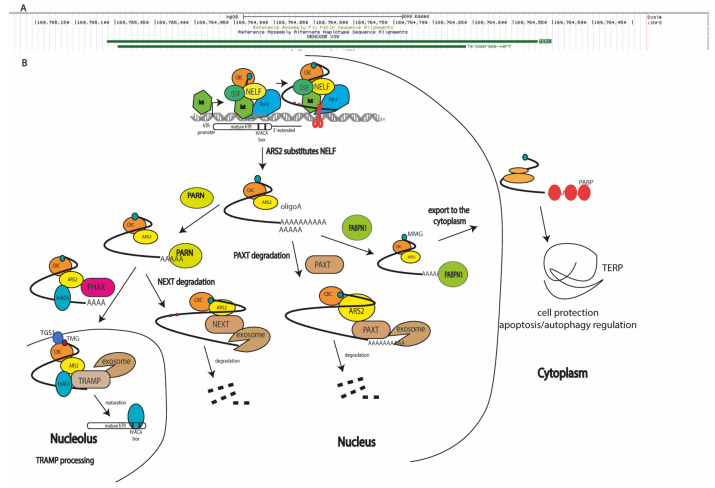
Biogenesis pathways of human telomerase RNA transcripts (hTERC): (**A**) two isoforms of hTERC presented in cells (https://genome.ucsc.edu accessed on 9 June 2022); (**B**) scheme illustrating biogenesis mechanisms of products transcribed from *hTERC* gene. Integrator (Int), DSIF, NELF, and cap-binding complex (CBC) assist RNA polymerase II to synthesize the primary hTERC transcript [14,27]. ARS2 substitutes NELF and coordinates the distinct, mutually exclusive complex assembly to process the primary transcript [29,30,31,32]. During processing, monomethylated (MMG), polyadenylated hTERP mRNA is protected by PABPN1, which is substituted by PABP and exported to the cytoplasm, where it is translated [18,27]. Oligoadenylated hTERC transcripts may be degraded by RNA exosomes attracted by the PAXT complex [27], or may be trimmed by PARN followed by degradation through NEXT-attracted RNA-exosomes [21], or transported by PHAX to the nucleolus [28] where it is hypermethylated (TMG) by TGS1 [16] and matured by TRAMP-mediated RNA exosome trimming [15,18] for assembly into the active telomerase complex.

Telomerase RNA is synthesized as a long precursor in Integrator-dependent mode [14], which is processed to obtain the mature RNA component of telomerase (451 nucleotides in length; see Figure 1A) [9]. Transcripts elongated up to 1000 nucleotides (Figure 1A) have been identified using next-generation sequencing (NGS) [15] and reverse transcription–polymerase chain reaction (RT-PCR) [16] techniques. Pathways of TERC processing (Figure 1) have also been reviewed in detail [17]. Oligoadenylated hTERC premature transcripts are trimmed by exosomes attracted through the interaction with adaptor proteins [15,18]. In the nucleoplasm, the nuclear exosome-targeting complex (NEXT) participates in the recruitment of exosomes to early unprocessed *hTERC* transcripts, resulting in degradation (Figure 1B). The shorter *hTERC* transcripts, containing several additional nucleotides at the 3′-end, are oligoadenylated by non-canonical poly(A)polymerase PAPD5 [19,20] and processed by poly(A)ribonuclease PARN [21,22], or by the TRAMP complex (Trf4/Air2/Mtr4p polyadenylation complex) [23] in the nucleolus or nucleoplasm, followed by transport to Cajal bodies [24]. Oligoadenylated *hTERC* precursors may be directly transported in Cajal bodies, where they are processed by PARN and TOE1 exonucleases [25] (Figure 1B). Finally, the matured hTERC is localized in Cajal bodies. The depletion of PABPN1 (poly(A)-binding protein nuclear 1) results in a decreased level of total *hTERC*, which is rescued when NEXT exosome components are co-depleted [18,26]. The depletion of the PAXT (poly(A) tail exosome targeting) complex involved in the degradation of polyadenylated RNAs through interaction with PABPN1 results in the accumulation of the unprocessed 3′-extended form of *hTERC* [26,27]. Polyadenylation is necessary for processing *hTERC* to the mature component of the telomerase complex. The impairment of this stage leads to the accumulation of unprocessed transcripts in the cytoplasm, where translation resulting in the synthesis of *hTERP* occurs (Figure 1B). It has been proposed that the gradually lengthened poly(A) tail acts as a sensor, and PABPN1 is preferentially recruited to *hTERC* precursors with longer poly(A) tails in order to prioritize their maturation [17].

However, PABPN1 can be substituted with PABP (poly(A)-binding protein) [26] to direct the export of long *hTERC* polyadenylated transcripts into the cytoplasm for translation. *hTER*C cytoplasmic localization in normal conditions has recently been determined [12,16]. Initially, the cytoplasmic localization of *hTERC* was observed when dyskerin or PARN were depleted and accumulation or defects in processing occurred. The co-depletion of exosome subunit RRP6 and cytoplasm decapping factor DCP2 restores the nuclear localization of *hTERC* under the condition of decreased levels of dyskerin or PARN [22]. The enzyme responsible for the polyadenylation of *hTERC* localized in cytoplasm has not yet been identified. Interestingly, the depletion of canonical PAPs decreases the hTERC level, providing an argument for the mRNA properties of *hTERC* (Figure 1B).

Co-transcriptional monomethylguanosine (MMG) modification occurs at the 5′-ends of transcripts synthesized by RNA-polymerase II. The MMG cap on precursor snRNAs recruits the cap-binding complex (CBC), the PHAX adaptor protein, and CRM1 for transport to the cytoplasm [28]. After hypermethylation by the trimethylguanosine synthase 1 (TGS1) of the MMG cap, snRNAs are reimported to the nucleus. Conversely, PHAX directs MMG-capped C/D-box snoRNAs to Cajal bodies where TGS1 hypermethylates the cap. The obtained trimethylguanosine (TMG)-capped C/D-box snoRNA recruits CRM1 in a CBC-independent manner, which is necessary for nucleolar localization [28]. The modification of the cap and the nuclear transport and export of *hTERC* is less well-investigated; however, it is known that TGS1 is responsible for the hypermethylation of MMG-capped *hTERC* (Figure 1B) and that the depletion of TGS1 leads to the cytoplasmic accumulation of *hTERC* (Figure 1B), where it is not associated with hTERT. Notably, the total level of *hTERC* transcript was increased in TGS1-depleted cells, without the redistribution of portions of 3′-end-extended and oligoadenylated forms of *hTERC* [16].

It has recently been demonstrated [27] that PHAX deficiency does not influence the level of mature *hTERC*. The co-depletion of the PAXT complex decreased the amount of mature and 3′-extended *hTERC* transcripts, and the co-depletion of TRAMP stimulated the accumulation of 3′-extended hTR transcripts, especially in the cytoplasmic fraction. We hypothesize that, in the absence of both the PHAX transport factor and TRAMP complex, the 3′-extended *hTERC* transcripts accumulate and are redirected to an alternative transport pathway which is inherent to mRNA.

Taking the details of processing and the localization of *hTERC* together, we can conclude that the *hTERC* gene produces two distinct types of molecules: the TMG-capped RNA component (451 nucleotides in length) of telomerase, which localizes in the nucleus and facilitates telomere synthesis in complex with *hTERT* in actively proliferated cells, and MMG-capped 3′-end-extended mRNA localized in the cytoplasm, which directs the synthesis of *hTERP* under unidentified conditions (Figure 1).

## 3. Structural Features of hTERC Responsible for Telomerase Activity

**Figure 2:**
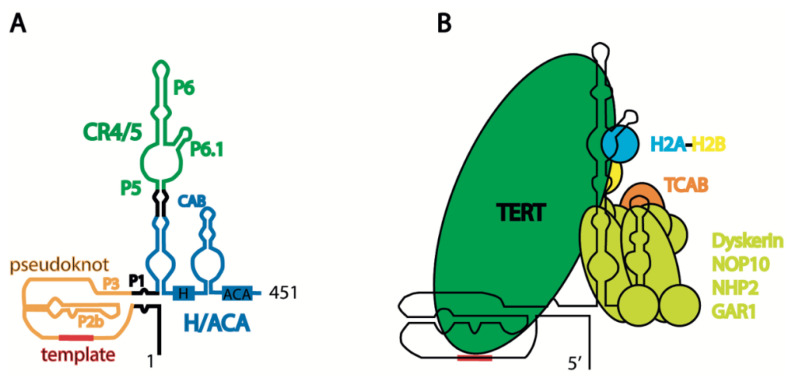
Schematic presentation of human telomerase structure: (**A**) secondary structure of mature human telomerase RNA hTERC; and (**B**) schematic view of human telomerase holoenzyme complex.

Considering the evidence that distinct forms of *hTER*C are present in cells, the structure of the mature telomerase component has been investigated in detail, where the object of investigation was TERC as a component of the ribonucleoprotein complex that provides the structural frame for telomerase. The telomerase complex structure has recently been reviewed in detail [33]. *hTERC* contains several structural domains essential for telomerase activity. The pseudoknot and H/ACA domains may reconstitute telomerase activity, even if they are expressed separately [34,35] (Figure 2A). Pseudoknot is important for binding *hTERT* and contains regions which possess a template for telomere synthesis (Figure 2B). The H/ACA domain, along with the NOP10, NHP2, dyskerin, and GAR1 proteins, forms the H/ACA complex [36], which blocks excessive degradation by exosomes and PARN, which trims the nascent transcript [18,21] (Figure 1A). The formation of a triple helix at the 3′-end of *hTERC* prevents its excessive degradation prior to binding the H/ACA complex [37]. The CAB-box of the H/ACA domain contains the signal for localization in Cajal bodies, as assisted by the TCAB1 protein [38] (Figure 2B). The association of the TCAB1 protein with the CAB-box promotes the formation of a tertiary structure in the CR4/5 region of *hTERC*, which is preferable for association with hTERT [39]. TCAB1 dysfunction prevents telomerase complex formation, leading to a decrease in telomerase activity and the shortening of telomeres in human embryonic stem cells [40]. Conservative regions 4/5 (CR4/5), consisting of P5, P6, and P6.1, are responsible for association with hTERT, and the P6.1 stem is cooperatively shaped by TERT and histone H2A-H2B, the presence of which has recently been revealed in the active telomerase complex [41] (see Figure 2). 

## 4. Human Telomerase RNA Structural Elements Form during Telomerase Complex Assembly

Structural investigations have mainly focused on the telomerase complex and telomerase RNA in the context of telomerase. Several investigations have been performed in vivo where the TERC structure was chemically probed in cellular lysates [42,43]. Much effort has been put into the investigation of the pseudoknot domain of *hTERC* by NMR [44,45]. Finally, cryo-EM structures of human telomerase and its complex with substrate have recently been reported [36,41]. Significant information has also been obtained from biochemical investigations of telomerase activity using mutant variants of the enzyme component. 

**Figure 3:**
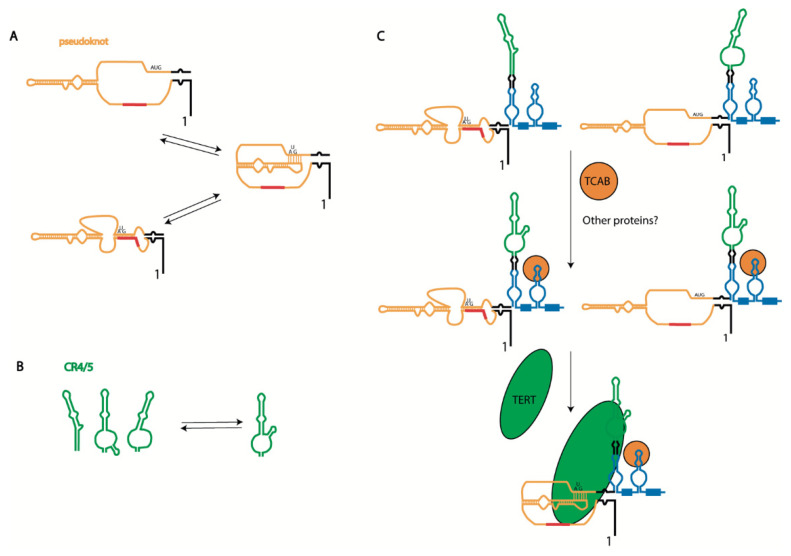
Biogenesis-driven human telomerase RNA structure formation: (**A**) alternative structures of pseudoknot domain of *hTERC*; (**B**) alternative structures of CR4/5 domain of *hTERC*; and (**C**) scheme illustrating *hTERC* structure formation during telomerase complex assembly. The primary synthesized *hTERC* transcript may be folded into two alternative structures [45,49]. Binding of TCAB1 facilitates folding of the P6.1 stem and CR4/CR5 domain and Cajal body localization [39], where TERT binding promotes pseudoknot formation associated with proper template region positioning with respect to the enzyme active center [42].

The search for genes which, through their dysfunction, are responsible for diseases characterized by short telomeres and senescence phenotypes—known as telomeropathies—has revealed various telomerase components and components involved in telomerase biogenesis. Such mutations predominantly affect the steady-state accumulation of *hTERC* [46]. Dyskerin mutations lead to the accumulation of *hTERC* in the cytoplasm or excessive degradation by exosomes during biogenesis (Figure 1); however, mutations in the *hTERC* sequence may affect telomerase activity without presenting an apparent impact on *hTERC* steady-state accumulation. The dysfunction of TCAB1 or mutations in the CR4/5 domain of *hTERC* disturb TCAB1-dependent tertiary structure formation, blocking the interaction with hTERT but not influencing the stability of *hTERC* [39] (see Figure 2B). Mutations in hTERC associated with the dyskeratosis congenita (DKC) disease include a shift in the equilibrium toward the hairpin structure due to pseudoknot destabilization (Figure 3A) [44]. Critical evaluations of the disease-relevant impact of *hTERC* variants not compromised for accumulation in vivo have been performed in several works [47,48], which revealed that disturbances to almost all stems presented in the *hTERC* structure impair telomerase activity. Substitutions that replace a canonical base pair with a wobble pair have a less severe effect than the most disruptive substitutions. Each mutation affecting the structure of the P2b and P3 stems of the pseudoknot domain (Figure 2A) caused the dramatic inhibition of telomerase activity; however, mutations in other regions of the pseudoknot domains did not affect telomerase activity significantly. Mutations in the CR4/5 domain, which is responsible for hTERT binding, also affected telomerase activity. The most dramatic influence was observed when mutations disrupted the structure of the P6.1 stem (Figure 2A) [47,48]. 

The telomerase pseudoknot domain may form a phylogenetically conserved hairpin, which is stabilized by a unique uridine helix (Figure 3A). A functionally important interconversion between the hairpin and pseudoknot conformations has been proposed [44]. The existence of the hairpin, formed as an alternative structure to the pseudoknot, was initially determined by NMR [44,49]. Several years later, functional mutational analysis of the pseudoknot structure in human telomerase RNA confirmed the importance of the pseudoknot structure for telomerase activity, both in vitro and in vivo [50]. Indeed, it has been demonstrated that mutations which abolish the P3 helix have an inhibitory effect on telomerase activity, which was reversed through compensatory mutations. It was concluded that the alternative hairpin structure does not maintain telomerase function, as destabilization of the intraloop J2b/3 base pairing did not affect the enzyme activity. Unfortunately, the effect of stabilization of J2b/3 intraloop pairing on telomerase activity was not investigated [50]. The existence of alternative structures of the pseudoknot domain of telomerase RNA in the ciliate Tetrahymena and yeast *Saccharomyces cerevisiae* strengthen the hypothesis about its evolutionary origin. An idea regarding the regulatory role of the P3 stem in active telomerase complex formation has recently been proposed and checked [51], and the data obtained suggest that the nascent *hTERC* transcript initially folds as an alternative hairpin, which is then remodeled into the more stable pseudoknot structure with time (Figure 3A). *hTERT* interaction could promote the refolding of *hTER*C. Interestingly, in this model of the alternative hairpin, the template region of *hTERC* is included in the stem stabilizing this structure (Figure 3A). The authors hypothesized that such a structure protects the single-stranded template region until the complex with hTERT is formed. Moreover, it was suggested that the order of the pseudoknot folding and interaction with *hTERT* is important for proper complex formation. According to this model, *hTERT* should bind the CR4/5 domain first, then promote the pseudoknot folding associated with correct template accommodation in the catalytic center of *hTERT* to produce the active enzyme; meanwhile, the interaction of *hTERT* with the pre-folded pseudoknot leads to incorrect template positioning in the enzyme active center, producing a non-functional telomerase complex.

Mutations disrupting the structure of the P6.1 stem of the CR4/5 domain of *hTER*C result in telomerase dysfunction and diseases related to telomeropathies. The P6.1 stem is an evolutionarily conserved structural feature of telomerase RNA that forms a three-way junction fold. Careful analysis of the structure of this region, performed using different techniques such as chemical probing, NMR, cryo-EM, and FRET, has demonstrated its structural heterogeneity in free *hTERC* and *hTERC* bound with hTERT [39,42,52,53,54] (see Figure 3B). The association of hTERT with the CR4/5 domain depends on the structure formed by the P6.1 stem, and mutations which destabilize its three-way junction structure prevent *hTERT* binding. 

Taken together, these structural data and mutational analysis may allow for the proposal of a model of telomerase complex association (Figure 3C). The native transcript is presented in several steady-state conformations [44,50,51,55]. The H/ACA-domain should be folded first, which occurs co-transcriptionally (Figure 1). The binding of H/ACA proteins protects *hTERC* from excessive exosome-driven 3′-end processing; however, the folding of *hTERC* requires specific interactions. TCAB1 binding is necessary for the P6.1 stem folding responsible for the association of the CR4/5 domain with *hTERT* [39]. Finally, the pseudoknot structure is formed after *hTERT* binding, which facilitates the positioning of the template region at the active center of the enzyme [42] (Figure 3C).

## 5. hTERC Structure and Localization Determine Its Function

Intuitively, it is expected that the mutations in *hTERC*—especially in domains involved in its association with *hTERT*—will affect telomerase activity. Many efforts have been undertaken to determine the intracellular localization of *hTERC*; however, the data obtained in different works show no unity, and the question regarding the natural localization of *hTERC* (and, moreover, that of its different isoforms) remains open. The localization of *hTERC* has been explored mainly in cancer cells and under conditions of the overexpression of mature *hTERC* capable of forming the complex with hTERT. It has been demonstrated that *hTERC* localizes in Cajal bodies only in the presence of hTERT [56], where the active telomerase complex assembles and interacts with telomeres. Telomerase RNA has been found to be localized in the nucleoplasm, nucleolus, Cajal bodies, cytoplasm, and mitochondria [12,16,22,56,57,58]. The functionality of the non-nuclear localizations of telomerase RNA is not yet clear. Nucleolar localization is necessary for *hTERC* processing or turnover, and it is thought that Cajal body localization promotes telomerase complex assembly and association with telomeres. However, it has been demonstrated that telomerase yields proper telomere synthesis even in cells with disrupted Cajal bodies [24].

Evidence for *hTERC* function outside of the telomerase complex has recently been reviewed [11]. It has been found that the mutant form of *hTERC* which is unable to form telomerase enzyme protects cells under an apoptosis-inducing treatment [59]. Analysis of the *hTERC* sequence has revealed that it contains the open reading frame that encodes for the protein named *hTERP* [12]. The existence of *hTERP* has been demonstrated clearly, and it has been shown that it protects cells under stress conditions and is involved in the regulation of autophagy and proliferation signaling pathways [13]. However, the position of the start AUG codon in the tertiary structure of *hTERC* is not convenient for ribosome binding and efficient translation (Figure 1). Data confirming the existence of multiple forms of native *hTERC* transcripts and alternative folding of the naked *hTERC* transcript provide additional arguments in favor of *hTERC* protein coding capacity. We hypothesize that the alternative structure of *hTERC*, distinct from the pseudoknot, may provide ribosome scanning and translation initiation abilities; the existence of alternative structures of *hTERC* are necessary to facilitate *hTERP* synthesis when hTERT is absent, and cells do not require the synthesis of telomere repeats.

We hypothesize that auxiliary factors regulate the biogenesis, structure formation, and function of native *hTERC* transcripts. The binding of additional RNA chaperones should stimulate specific structure stabilization and transcript localization. We speculate that the structure of *hTERC* should be subject to regulation by some yet-undetermined factors or intracellular conditions (e.g., pH, ionic strength) in order to determine the fate of particular transcripts—either to become part of the telomerase complex and function in the nucleus, to be exported to the cytoplasm to meet the ribosomes, to be transported to mitochondria, or to be degraded.

## 6. Conclusions

Taken together, the available data concerning the assembly of the telomerase complex and the multiple mechanisms involved in TERC processing suggest that the function of TERC transcripts depends on their structure, bound proteins, and the exact intracellular localization of the final product of biogenesis. Novel data have uncovered new forms of *hTERC* transcripts and other non-telomerase functions of *hTERC*, opening perspectives for further investigations of the role of telomerase RNA transcripts in cellular functioning, survival, metabolism switching, and other aspects of cellular homeostasis.

## Data Availability

Not applicable.
